# Parameter extraction and selection for a scalable N-type SiC MOSFETs model and characteristic verification along with conventional dc-dc buck converter integration

**DOI:** 10.1371/journal.pone.0277331

**Published:** 2023-01-13

**Authors:** Hassan Khalid, Saad Mekhilef, Marif Daula Siddique, Addy Wahyudie, Mahrous Ahmed, Mehdi Seyedmahmoudian, Alex Stojcevski

**Affiliations:** 1 Power Electronics and Renewable Energy Research Laboratory (PEARL), Department of Electrical Engineering, University of Malaya, Kuala Lumpur, Malaysia; 2 School of Science, Computing and Engineering Technologies, Swinburne University of Technology, Hawthorn, VIC, Australia; 3 United Arab Emirates University, Al Ain, United Arab Emirates; 4 Taif University, Taif, Saudi Arabia; Jamia Millia Islamia A Central University, INDIA

## Abstract

Most silicon carbide (SiC) MOSFET models are application-specific. These are already defined by the manufacturers and their parameters are mostly partially accessible due to restrictions. The desired characteristic of any SiC model becomes highly important if an individual wants to visualize the impact of changing intrinsic parameters as well. Also, it requires a model prior knowledge to vary these parameters accordingly. This paper proposes the parameter extraction and its selection for Silicon Carbide (SiC) power N-MOSFET model in a unique way. The extracted parameters are verified through practical implementation with a small-scale high power DC-DC 5 to 2.5 output voltage buck converter using both hardware and software emphasis. The parameters extracted using the proposed method are also tested to verify the static and dynamic characteristics of SiC MOSFET. These parameters include intrinsic, junction and overlapping capacitance. The parameters thus extracted for the SiC MOSFET are analyzed by device performance. This includes input, output transfer characteristics and transient delays under different temperature conditions and loading capabilities. The simulation and experimental results show that the parameters are highly accurate. With its development, researchers will be able to simulate and test any change in intrinsic parameters along with circuit emphasis.

## Introduction

The demand for renewable source of energy has been greatly increased as compared to conventional non-renewable energy sources like fossil fuel and coal. One of the main reasons for shifting to renewable resources is that they are carbon emission-free and economical. With the wide development in power electronic converters, the Joint Research Center published a report which revealed that almost 133.7 billion USD were invested in 2016 for the PV installation to support a sustainable environment [1]. The widespread of renewable energy mainly rely upon the power converters’ conversion efficiencies. Since energy extracted from multiple resources like soler cells and hydro stations exists in DC or AC form, these power converters (AC, DC) become very important. These days, DC-DC power applications are widely explored in both low and high power applications [2–4]. These DC-DC converters are integrated with renewable energy sources (RESs), microgrids (MGs), high frequency (HF), high voltage direct current (HVDC) and electric vehicle (EV) like similar applications [5–8]. The driving element of these applications is mostly a MOSFETs.

On the other hand, these power electronics switches are also regarded for utilizing and managing the available energy significantly better. Presently, about 87% of all power electronic devices use silicon (Si) technology. But the conventional material used to develop MOSFETs has few drawbacks. One of its main drawbacks is that the large portion of the energy generated by the generation units is majorly consumed by the semiconducting device itself. The most commonly observable example is DC/AC vice versa converter losses. Owing to these problems, Silicon Carbide (SiC) MOSFETs have emerged as the best solution [9].

The silicon carbide (SiC) based devices are highly preferred due to fast switching, low switching losses, and as compared to the conventional silicon-based devices, exhibit low ON-state resistance, has a wide bandgap (WBG), has high breakdown voltage characteristics [10, 11], and can operate very efficiently even in extreme temperature conditions (max 200°C). These devices are very difficult to manufacture and require special environmental conditions to process them and few techniques like silicon-via-technology are very deeply discussed in [12].

To get the desired characteristic results, it is necessary to have a generic programmable model that can give access to the user to set the desired intrinsic and extrinsic parameters. To realize the benefits of SiC MOSFET modeling, the most powerful design hinges on the availability of the model. In industry, these devices are at first designed by using a regressive approach and device dynamics are further processed using Technological Computer-Aided Design (T-CAD) software and are finalized for the fabrication section. Since the developing process is highly sensitive to the environment and involves a bulk of cost investment. Therefore, the simulation program is a key factor for the design process as mentioned by Victory et al. [13], and by He et al. [14]. Circuit designers simulate the model parameters to reduce the gaps between TCAD and fabrication. But the parameter extraction and its selection to obtain the device characteristics for a specific model by its user have always been quite challenging. It also requires prior knowledge of material and device properties to develop any required SiC MOSFET model for a specific application. Time constraints also make it impractical to develop it other than go for the hit and trial method.

The model so far has been contributed by the following researchers. Blake W. Nelson et al. in [15] has deeply summarized the brief history of level 1 SiC MOSFET models and has compared the computation time of Physics, Semi-Physics, and behavioral models. Mukunoki et al. presented the physics-based model and considered channel mobility as a major factor for simplifying computational complexity [16]. However, a high level SiC MOSFET model is not yet contributed and this is the main contribution of this paper.

Mukunoki et al. in [17], presented the analytical model and showed the dependence of *V*_*GS*_ over gate and drain capacitances as *C*_*GS*_ and *C*_*GD*_. Most of the presented work focuses on improving the model I-V characteristics. Similarly, for improving model characteristic, a highly accurate and least complex design with reduced simulation and processing time is required. McNutt et al. in [18], presented 2kV/5A model based on the Hefner modeling technique, but his model implementation is complex. Later on, Jouha et al. in their work [19] extended the work of McNutt by expanding the concept of channel length modulation (*λ*). Chen in [20], improved the transient analysis of the device by relating them controlled by considering gate to source capacitance (*C*_*GS*_) as a non-linear junction. This nonlinearity usually arises due to nonlinear variation in the drain to source (*V*_*DS*_) voltage, the capacitance and channel width decrease with the increase in *V*_*DS*_. The nonlinear behavior was later on extended by Duan et al., who suggested improving the device behavior by extracting junction overlapping capacitances [21]. Now the point came when junction temperature became a quiet concern. The method proposed by Scott in [22] is parameter extraction using the Levenberg Marquardt (L-M) algorithm for temperature variation. Then a linear regression method is employed to estimate the most precise parameters. This method has a few objections. At first, it offers a complex iterating method that consumes time. Secondly, it is confined to only one device and lastly, it does not propose any impact of changing the length and width of the channel. Although it also provides the least information for evaluating gate to drain side capacitance (*C*_*GD*_) or gate to source capacitance (*C*_*GS*_). Similar literature work was presented by Jinfeng Liu and et al. [23] to extract parameters for switch module packaging applications using equivalent models, impedance matching method, and by using matrix extraction method. These methods were very simple, were limited to few applications and offered a limited range of valid parameters and were limited to selective users. In [24], Marcello Ciono and et al. contributed to gate driving voltage by stating *R*_*DSON*_ dependence on (*V*_*GS*_−*V*_*TH*_) and later on it became a reason for casing parameter drift.

The simulators used by Nutt in [18], Chenn in [20], Duan in [21], and Scott [22] can propose changes to only terminal resistances and extrinsic capacitances. But for high-level parameter modeling of SiC MOSFET simulating model requires both device and semiconducting material specifications. The device ratings can be easily obtained from the manufacturer’s datasheet. However, the intrinsic details are calculated based on material physics.

In this paper, we came up with a novel technique for parameter extraction for any scaleable SiC MOSFET device. The other contribution of this paper is to propose a parameter selection strategy based on the graphical mapping without causing any convergence issue. For this purpose Shicman—Hodges model is used and level 3 parameters are extracted for SiC NMOS. The model thus developed is flexible to almost nano-scale dimensions. The modeled parameters are verified by SiC MOSFET C2M0025120D manufactured by CREE which can transfer 1200V/ 90A, drain to the source with only 23 mΩ ON resistance. To further test the extracted parameters a small-scale DC-DC buck converter with 1MHz switching frequency and 97% efficiency was verified using both simulation and practical experimental setup. This extraction method is highly accurate.

The methodology of this work is three folds: device specifications from manufacturer datasheet, parameter mapping, and its selection using the proposed method. This paper is organized as follows; section 2 discusses the parameter extraction of the SiC MOSFET, section 3 implements the buck converter, while section 4 presents the critical discussion and comparisons. In the end section, 5 concludes the whole discussion.

Some of the highlights of this article are summarized as follows

- This article successfully extracts the required parameters to develop programable SiC MOSFET
- The steps involved in extracting the required information from the given SiC MOSFET have been illustrated in a sequential manner
- It is assumed that the device operates at average room temperature
- To verify the proposed method, the programming model is implemented to form a buck converter, and both simulation and experimental results are closely related.

## Parameter extraction methodology

**Fig 1:**
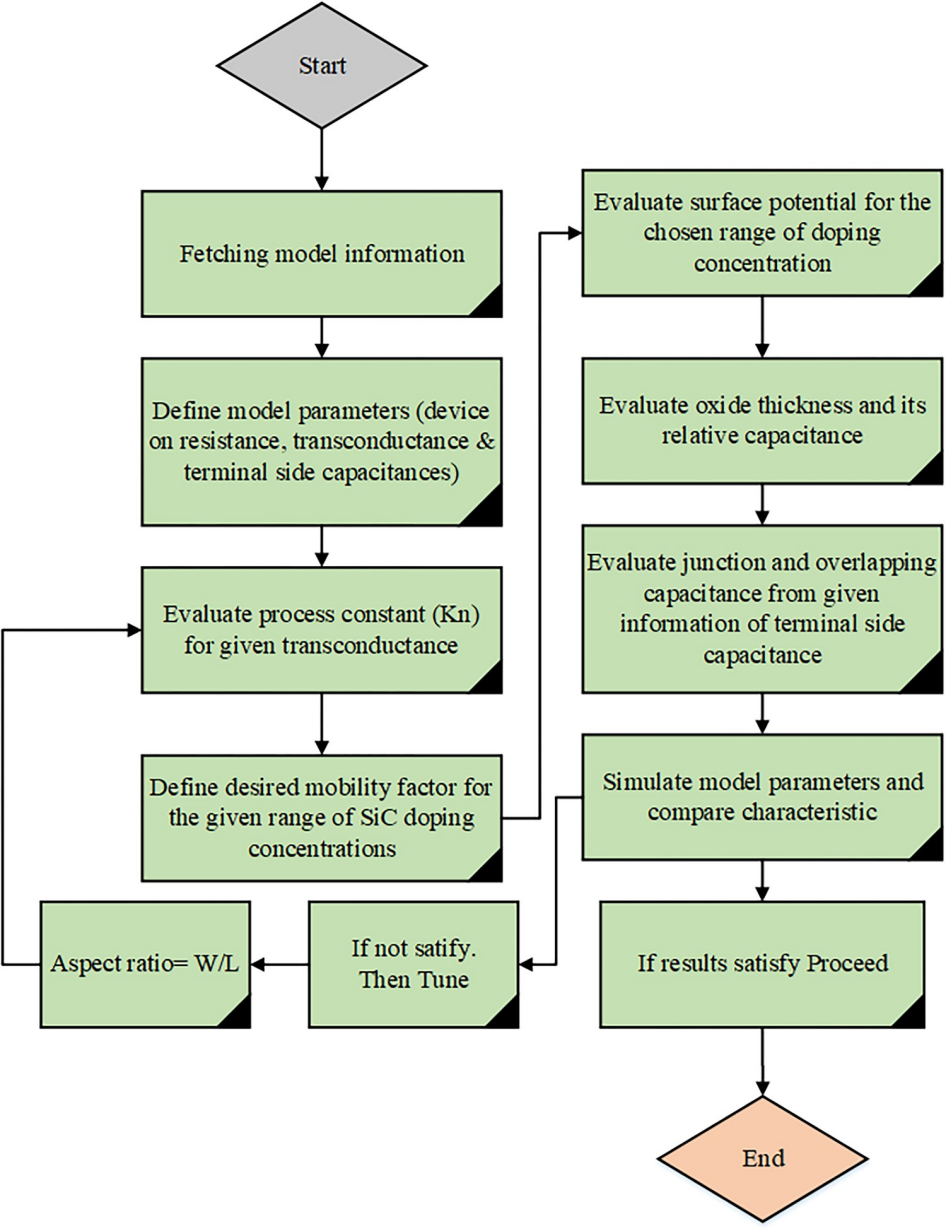
Process flow diagram for parameter extraction.

This section presents the methodology for extracting device parameters. It is three fold: defining a set of device intrinsic and extrinsic device variables, extracting useful information from the manufactured device, and obtaining level 3 nmos scaleable parameters. The proposed method is applied to C2M0025120D manufactured by CREE to validate the extracted parameters which satisfy our model. However, any model of SiC Mosfet parameters can be extracted by using following sequential process illustrated in Fig 1.

### Process constant (*K*_*n*_)

To state the steady-state characteristics of NMOS, the Shicman—Hodges drain current model has been adopted which offers fewer iterations in simulation and therefore consumes less time [25]. The MOSFET drain to the source channel conducts current when the applied voltage between gate and source terminal (*V*_*GS*_) is higher than its threshold level. For the fixed value of (*V*_*GS*_), the increase in drain current and drain to source voltage (*V*_*DS*_) is linear which defines the value of process constant (*K*_*n*_). However, this does not completely model channel conductance (*g*_*m*_) for the entire region. By using of small-signal modeling tool, we are able to model chanel resistance of both linear and non linear side. the ratio of small-signal drain current (*i*_*d*_) to the small-signal gate to source voltage (*v*_*gs*_)

$$g_{m}=\frac{\partial i_{D}}{\partial v_{gs}}$$ (1)

And now, using the small-signal model in the saturation region, the NMOS process constant (K_n_) can be related to channel transconductance as given in (2) [26].


$$g_{m}=\sqrt{\frac{{2K}_{n}W}{L}I_{D}}$$ (2)

In the saturation region, the MOSFET drain current becomes almost constant the drain current process constant (*K*_*n*_) and modulation index (*λ*) in (3) by Duan in [21].


$$I_{D}=0.5K_{n}(1+\lambda V_{DS}){\left(V_{GS}-I_{D}R_{s}-V_{th}\right)}^{2}$$ (3)

Substituting Eq (3) in (2) and setting the modulation index to zero for the sake of simplicity. We can relate channel conductance and drain to source voltage. Where (β) is called channel transconductance is called device transconductance, as in (4).


$$g_{m}=\beta V_{ds}$$ (4)

Using the Eqs from (1), (2), (3) and (4), the value of device transconductance and process constant for C2M0025120D are evaluated as 15.7*S*/*V* and 5.55*A*/*V*^2^ respectively. At this point, the aspect ratio is assumed to be unity.

### Mobility model

The Lombardi mobility model [27] best describes the MOSFET device by considering acoustic phonon (*μ*_*AC*_), surface roughness scattering (*μ*_*SR*_), coulomb scattering (*μ*_*C*_) and the scattered traps that occurred at (*μ*_*C*_). The Coulomb scattering mainly occurs due to the fixed oxide charge and the charges at the surface. At the low temperature, the Coulomb charge dominates the electron mobility and usually occurs in the weak inversion region of the SiC device. Therefore in this region, the inversion layer mobilities are defined by the sum of bulk mobility and the mobilities defined above and are mentioned by Eq (5)

$$u_{inv}={\left[\frac{1}{\mu_{B}}+\frac{1}{\mu_{AC}}+\frac{1}{\mu_{SR}}+\frac{1}{\mu_{C}}\right]}^{-1}$$ (5)

In the above (5), the Coulomb charge can be explained in terms of inversion charge (*Q*_*inv*_) defined by (7) and located at any point *y in a* cartesian coordinate system and perpendicular to the surface. Assuming the fixed charge distribution, the *μ*_*C*_ is related by the following equation

$$\mu_{C}=\frac{1}{Q_{trap}}T^{\alpha }Q_{inv}^{\beta }$$ (6)

$$Q_{inv}=\int_{0}^{\phi_{S}}\frac{n(\phi )}{E(\phi )}d\phi$$ (7)

Where T is a temperature, *α* is a temperature coefficient and *β* is an aspect ratio. The bulk mobility is described by Eq (8)

$$\mu_{B}(N)=\mu_{min}+\mu_{o}{\left(1+\frac{N}{N_{C}}\right)}^{\alpha }$$ (8)

**Fig 2:**
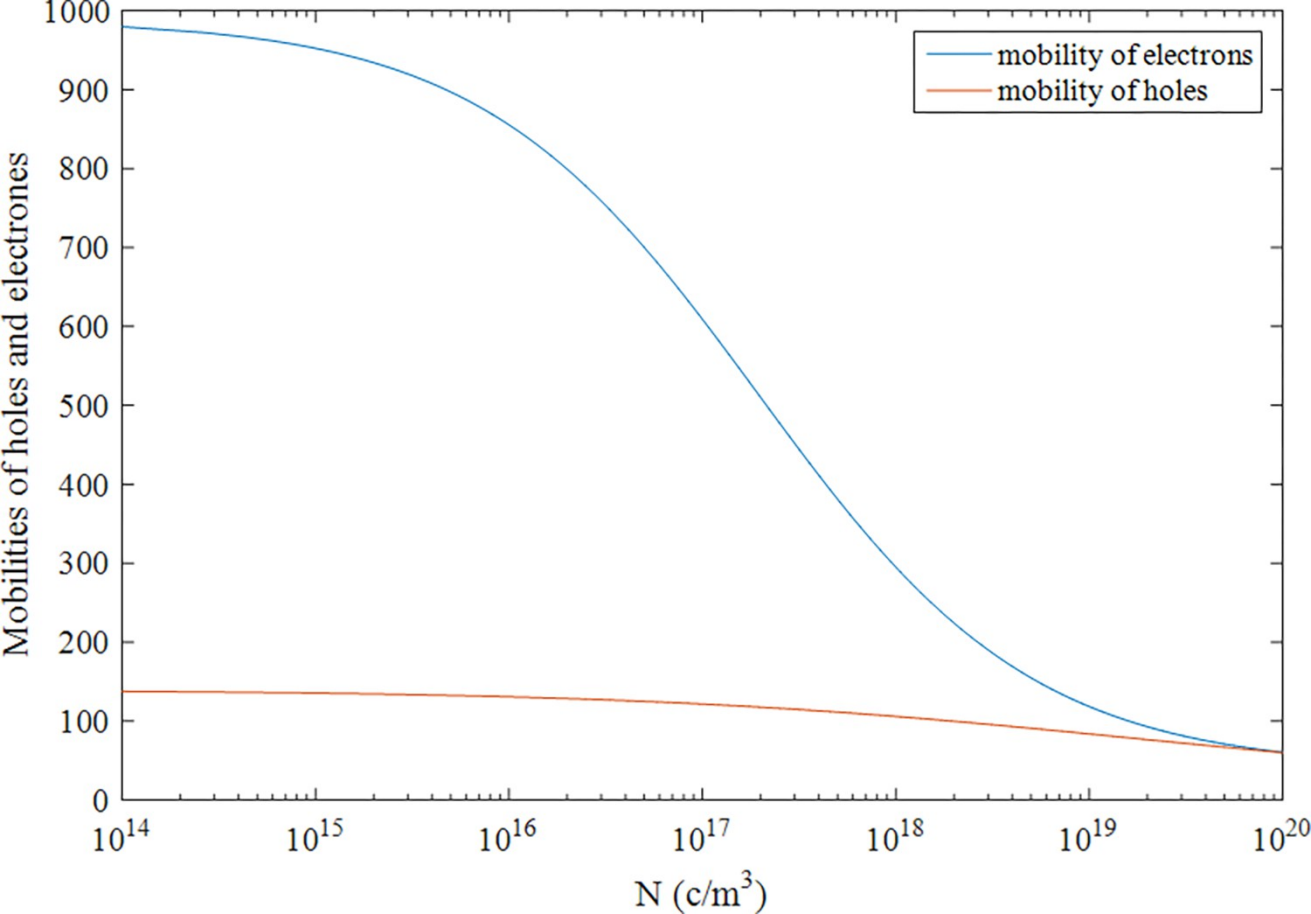
Electrons and holes mobility as a function of doping concentration.

Here, μ_0_ is called the mobility factor. For silicon carbide, the maximum and minimum range of electrons and holes mobility is given by (40–987) *cm*^2^/*Vs* and (15.9–140) *cm*^2^/*Vs* respectively. This mobility is a function of doping concentration *N*_*D*_. At low values of doping concentration, these values are almost independent of the mobility factor because the ionized impurity is much lower than lattice scattering, while the amount becomes considerable for the high value of concentrations. The carrier mobility in terms of doping concentration is given by Caughey–Thomas Eq (6) from Silicon and Silicon-Carbide Power MOSFETs by Kazimierczuk in [28]. In the above equation, *N = N*_*A*_ *+ N*_*D*_ depending upon holes or electrons and *α* = 0.34 and 0.61 as by Kazimierczuk in [28] for holes and electrons, respectively. The mobility as a function of their respective doping is shown for its entire region is illustrated in Fig 2.

The *u*_*inv*_ from (5) and inversion charge from (7) can be used to evaluate sheet conductance mentioned by (9).


$$\sigma_{s}=q\mu_{inv}|Q_{inv}|$$ (9)

In [29], authors have resolved sheet resistivity (*R*_*s*_) and Hall coefficient (*R*_*H*_) in terms of conductivity tesor components *σ*_*xx*_ and *σ*_*xy*_ in Eq (10). This contains information regarding all the carriers present in any sample.


$$\sigma_{xx}=\frac{R_{s}}{R_{s}^{2}+R_{H}^{2}};\sigma_{xy}=\frac{R_{H}B}{R_{s}^{2}+R_{H}^{2}}$$ (10)

This lead to the calculation of the overall Hall scattering factor (*r*_*H*_), which is defined by the ratio of Hal effect-mobility (*μ*_*H*_) and mean mobility (*μ*_*D*_). The Hall scattering is evaluated while assuming that the carrier mobility is least affected by the magnetic field. From Fig 2, fixing the values of mobilities at any concentration level will give the overall sheet conductance.

### Surface potential

This parameter is a function of bothe acceptor and intrinsic dopping. It is a voltage at the surface of silicon carbide intrinsic carrier and is given by the following

$$\phi_{s}(N_{a},N_{i})=2\frac{K_{B}T}{q}ln\left(\frac{N_{a}}{n_{i}}\right)$$ (11)

Where *K*_*B*_ = 1.38×10^−23^*JK*^−1^ is a Boltzmann constant and charge *q* = 1.6×10^−19^*C*. Here, *n*_*i*_ is an intrinsic carrier concentration and is responsible for limiting the operating temperature of the device. The intrinsic carrier concentration is directly proportional to material temperature, and for normal room temperature, the intrinsic carrier concentration is almost small as compared to *N*_*a*._ For silicon carbide intrinsic carrier concentration is given by following expression as in [30].


$$n_{i}=\sqrt{N_{c}N_{v}}e^{\frac{E_{g}}{2kT}}$$ (12)

**Fig 3:**
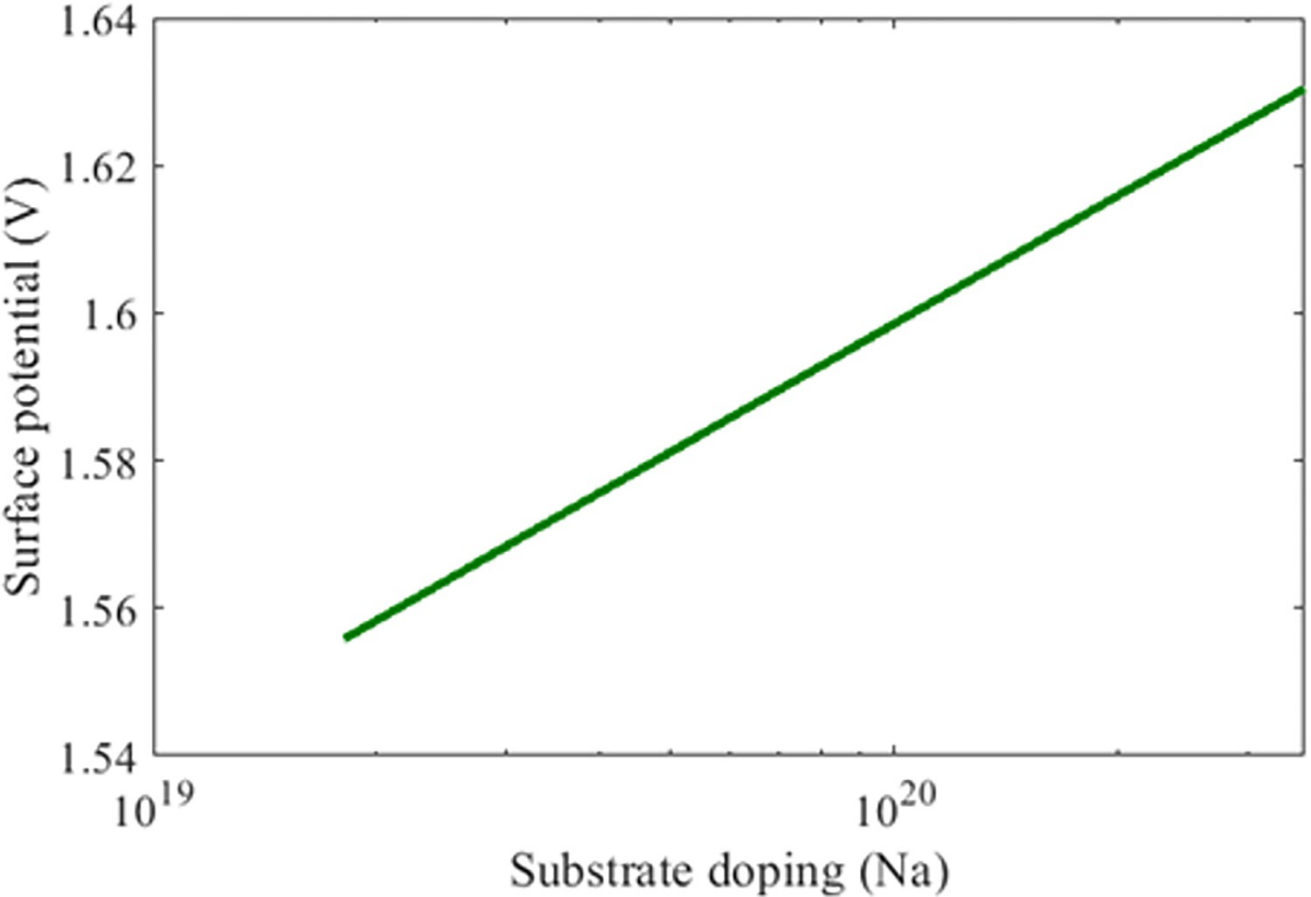
Sic nmos surface potential as a function of substrate doping.

Where *N*_*c*_ and *N*_*v*_ are doping concentration of conduction and valance band. By substituting the values for the SiC-based MOSFET can be simulated for the entire region and is illustrated in Fig 3. Based on the previous constraints the *ϕ*_*s*_ = 1.7×10^−8^*cm*^−3^.

### Oxide capacitance (C_ox_)

The bulk charge mobilities defined in the previous section and together with oxide thickness capacitance (*C*_*ox*_) defines the process constant of the device and is related by [13] and is illustrated in Fig 3A

$$K_{n}=\mu_{eff}C_{ox}$$ (13)

**Fig 4:**
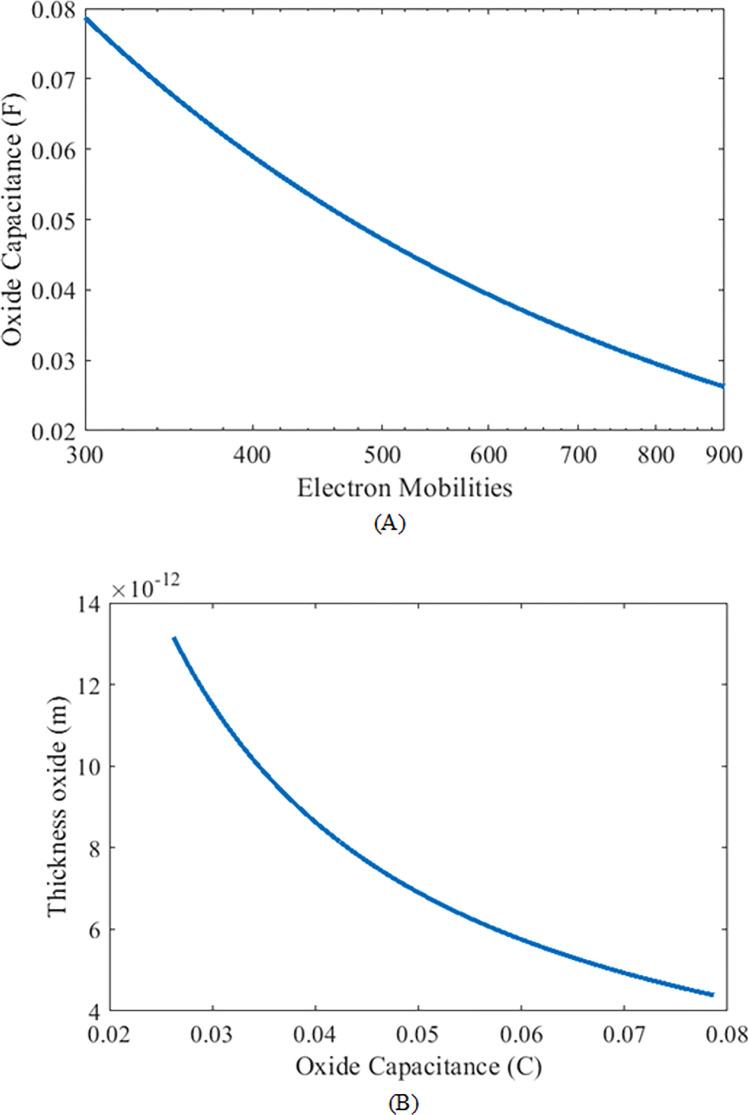
(A) Oxide capacitance as a function of electron motilities. (B) Thickness oxide as a function of Oxide capacitance.

In Fig 4A and 4B, the data is evaluated for the entire aspects of SiC MOSFET. It can be analyzed that as the thickness oxide of the channel decreases, more electrons get passage to pass easily and the oxide capacitance also decreases with an increase in *t*_*ox*_.

### Thickness oxide (*t*_*ox*_)

The metal oxide semiconductor (MOS) is divided into layers from metal to semiconductor; between metal and substrate, there is a layer of oxide with a specific thickness called thickness oxide (t_ox_). The length of thickness oxide varies as the gate voltage varies and is properly selected for given oxide capacitance related by following

$$t_{ox}(C_{ox})=\frac{\varepsilon_{ox}}{C_{ox}}$$ (14)

Where, $\varepsilon_{ox}=\varepsilon_{r(SiO_{2})}\varepsilon_{o}$ is a dielectric constant and the insulating medium used between the top and bottom plates of MOSFET is made of silicon dioxide. Based on the information extracted from the body bias coefficient, the oxide capacitance to oxide thickness can be related as shown in Fig 4B.

### Body bias coefficient (γ)

**Fig 5:**
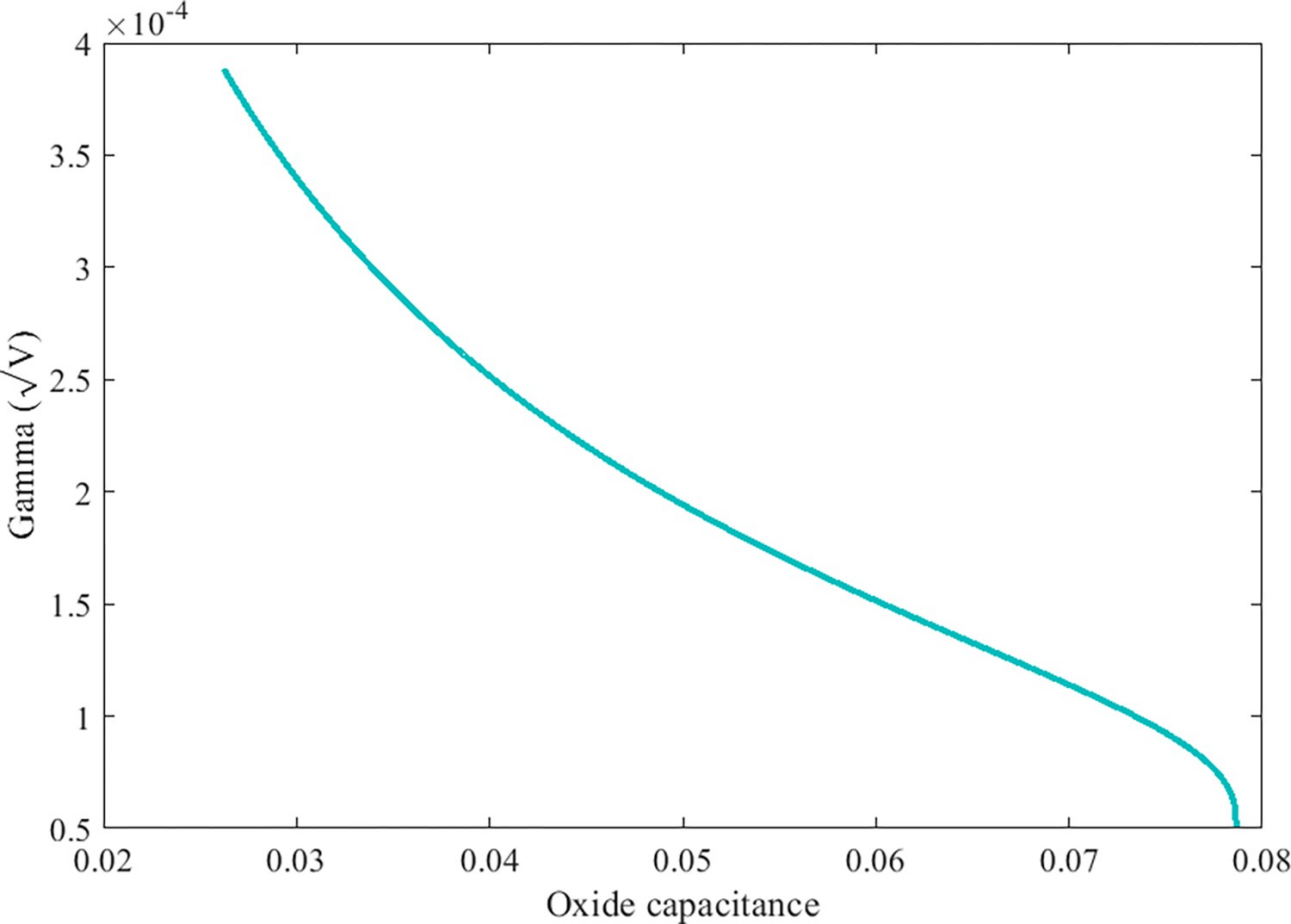
Variation in body bias coefficient by varying oxide capacitance.

The body bias coefficient (*γ*) varies both as a function of acceptor doping (*N*_*A*_) and oxide capacitance and is related by (15). Based on the ranges defined in the previous section for SiC MOSFET. We can have a clear idea about the variation in body bias coefficient factor and illustrated in Fig 5.


$$\gamma (C_{ox},N_{A})=\frac{\sqrt{2q\varepsilon_{ox}N_{A}}}{C_{ox}}$$ (15)

This factor plays a major role in controlling the threshold value of SiC MOSFET. It is set to have a four-terminal device, but the fourth terminal as the body bulk terminal, is connected to the body substrate. This is because the potential difference between source and body voltage highly affects the threshold voltage. This also makes it behave as a second gate and largely helps to control the transistor on-off state.

### Junction capacitances

**Fig 6:**
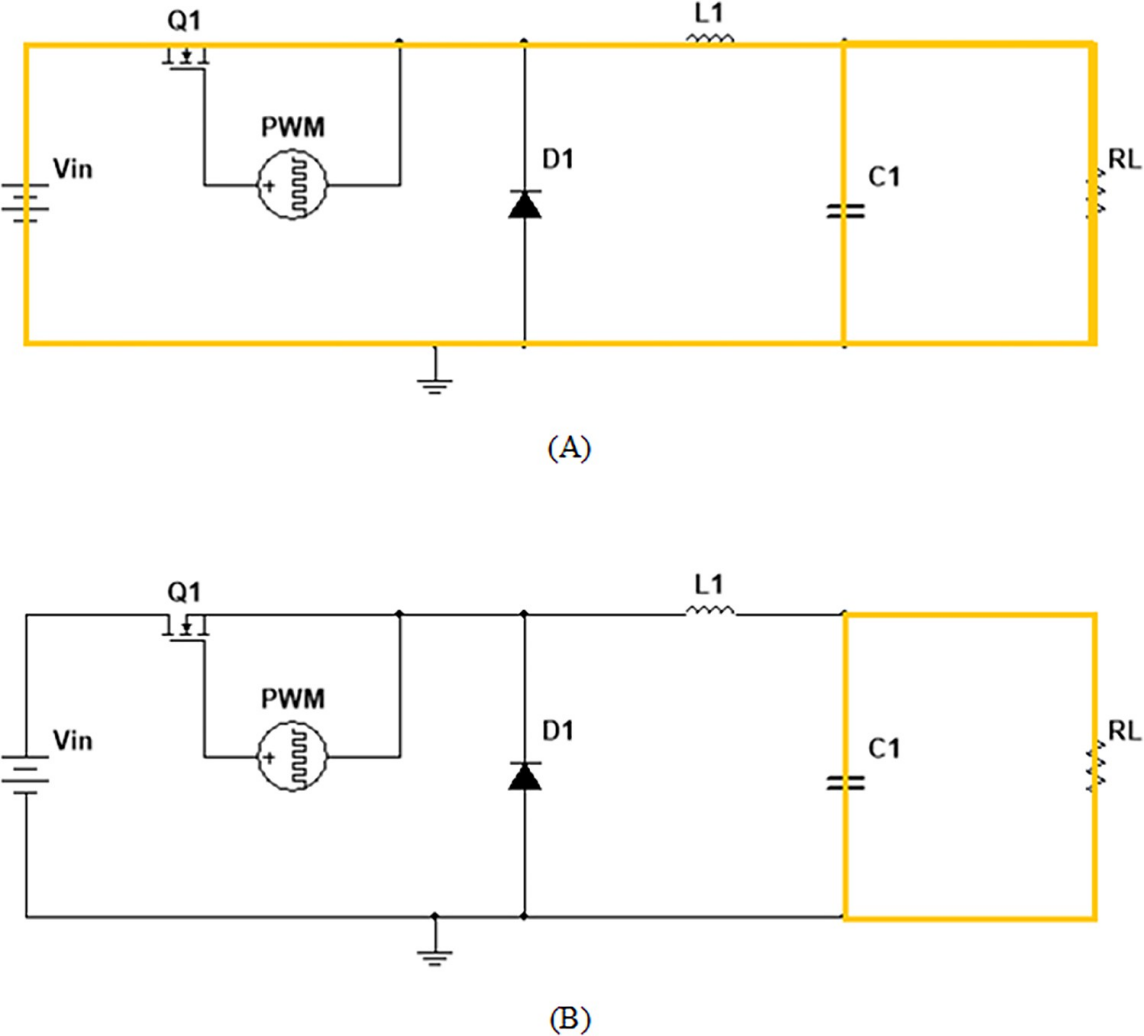
SiC NMOS Buck converter scheme includes (A) switch turns on (B) switch turns off and diode ***D*** conducts.

Consider the capacitance arrangement in Fig 6 according to the S—H model. The junction capacitance is equivalent to the capacitance between the drain and source and it varies as a function of drain to source voltage and is given by following.


$$C_{DS}(V_{DS})=C_{j}=C_{jo}{\left(1+\frac{V_{DS}}{V_{bi}}\right)}^{-m}$$ (16)

Where *C*_*jo*_ has the same value that of *C*_*DS*_ at *V*_*DS*_ = 0*V* and *V*_*bi*_ = 0.69 for the typical SiC-based MOSFET. From Fig 6 the input side, output side, and reverse side capacitance can be modelled by using (17), (18), and (19), respectively as (*C*_*iss*_), (*C*_*oss*_) and (*C*_*rss*_).

All the necessary parameters are taken as a base reference from the CREE manufacturer [31].


$$C_{iss}=C_{GS}+C_{GD}$$ (17)

$$C_{oss}=C_{GD}+C_{DS}$$ (18)

$$C_{rss}=C_{GD}$$ (19)

In this section, all the required parameters have been extracted using S—H as a base model. After this, these parameters are mapped graphically to form a mesh like structure so that any parameter could be selected accordingly. In Table 1 one set of extracted parameters for C2M200120D have been summarized. However, these can be easily modiefied for any aspect ratio or intrinsic parameter based on above principles. The parameter key selection is by selecting device transconductance, which in return interlinks thickness oxide, oxide capacitance, surface potential, and doping concentrations. In modeling, dynamic characteristics of the device, inter-electrode capacitance are modeled based on the static characteristics.

**Table 1:** Sic level 3 nmos model extracted parameter.

No.	Parameter	Symbol	Value	Units
1.	Acceptor doping	*N*_*a*_	2.84x1019	cm-3
2.	Body bias coefficient	*γ*	0.005485	$\sqrt{V}$
3.	Channel length (adjustable)	L	10*μ*	m
4.	Channel resistance	*R*_*DS*_	25	*m*Ω
5.	Channel width (adjustable)	W	10*μ*	m
6.	Drain to source side capacitance	*C*_*DS*_	205	pF
7.	Electron mobility	*μ*_*o*_	600	cm2/Vs
8.	Gate to bulk side overlapping capacitance	*C*_*GB*_	0.0277	F
9.	Gate to drain side capacitance	*C*_*GD*_	15	pF
10.	Gate to drain side overlapping capacitance	*C*_*GDO*_	0.46216	mF
11.	Gate to source side capacitance	*C*_*GS*_	2773	pF
12.	Gate to source side overlapping capacitance	*C*_*GSO*_	0.46216	mF
13.	Input side capacitance	*C*_*iss*_	2788	pF
14.	Junction capacitance	*C*_*Jo*_	0.04.035	nF/m2
15.	Maximum saturation velocity	*V*_*max*_	638.7x104	m/s
16.	Mobility modulation	*λ*	0.02	V-1
17.	Output side capacitance	*C*_*oss*_	220	pF
18.	Oxide Capacitance	*C*_*OX*_	0.009266	F/cm2
19.	Process constant *K*_*n*_	*K*_*n*_	5.55	A/V2
20.	Reverse side capacitance	*C*_*rss*_	15	pF
21.	Parallel internal gate resistance	*R*_*Gp*_	25	Ω
22.	Surface potential	*ϕ*_*s*_	1.567	V
23.	Thickness oxide	*t*_*Ox*_	3.729x10-7	μm
24.	Threshold voltage	*V*_*to*_	2.5	V

## Buck convertor implementation with proposed model parameter

Although much of the work has been presented in [32], this work uses a conventional DC-DC buck convertor model to verify the proposed scheme of parameter extraction. The parameters used in the simulation are summarized in Table 1.

The conventional buck converter operates in two modes. It consists of SiC MOSFET, a diode (*D*), an inductor (*L*), and a resistive load (*R*_*L*_). Solving circuits (Fig 6A and 6B) for interval *t*_1_ and *t*_2_, peak to peak ripples in current for the inductor is given by the following equation

$$V_{in}=V_{R}+L\frac{di}{dt}=V_{R}+L\frac{\Delta i}{\Delta t}$$ (20)

The total period is the sum of both transitions as in the following equations

$$T=t_{2}+t_{1}=\frac{1}{f}$$ (21)

$$t_{1}\frac{{\Delta i}_{L}}{V_{in}-V_{R}}$$ (22)

$$t_{2}=\frac{{\Delta i}_{L}}{V_{R}}$$ (23)

The capacitor value for controlling peak to peak ripples in voltage can be chosen by the following equation:

$$V_{c}=\frac{1}{C}\int_{0}^{\frac{T}{2}}i_{c}dt$$ (24)

$$\Delta V_{c}=\frac{1}{C}\int_{0}^{\frac{T}{2}}{\Delta I}_{c}dt$$ (25)

The peak to peak ripple voltage can be controlled by the capacitor by the following equation:

$$\Delta V_{c}=\frac{V_{in}D(1-D)}{8LCf_{sw}^{2}}$$ (26)

In the above equation, D is called the duty ratio which is the ratio of switch turn-on time to the total period. Putting (26) in (25) and after simplification, we get the peak to peak ripple current as in the following equation:

$$\Delta I=\frac{V_{in}D(1-D)}{f_{sw}L}$$ (27)

The experimental results and their discussion is presented in the next section.

## Results and discussion

The output characteristics of our proposed model have been validated by inserting all the parameters extracted from Table 1 and are exactly entered in the SPICE model directory and are compared to the characteristic curves with that of the C2M0025120D model data sheet [33]. In this section, the proposed model is first analyzed on behalf of its input transfer characteristics. After this, the output transfer characteristics are obtained from the proposed model at different values of the gate to source voltage and under different temperature conditions using SPICE. In the next test, the proposed model transient analysis is performed and it is observed that by changing the external gate resistance, the device transients are also changed. All the results obtained from these analyses are also discussed for the datasheet of the MOSFET model and it will be shown that the parameter extraction method proposed in section 2 is quite accurate.

**Fig 7:**
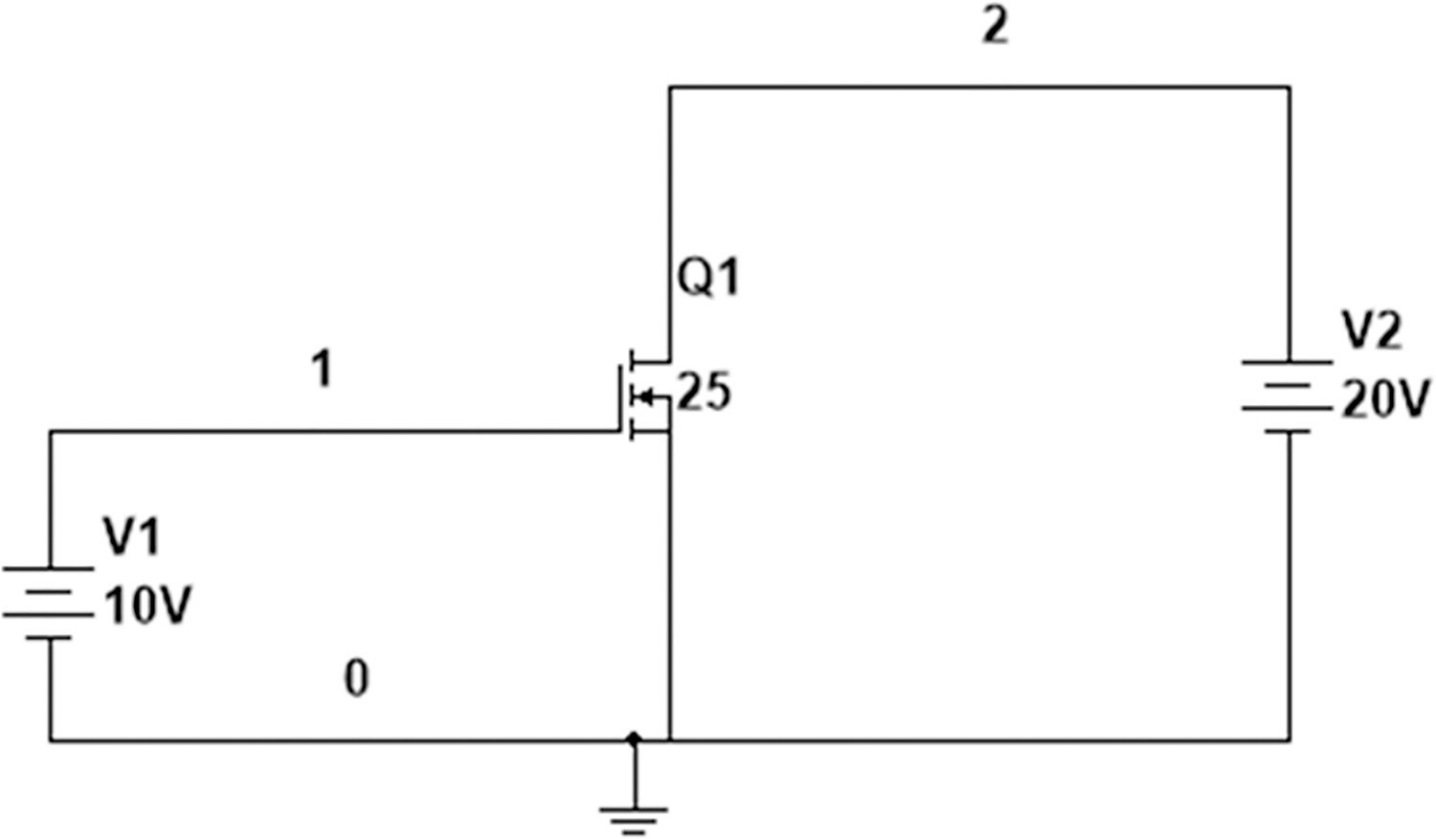
Schematic diagram of input transfer characteristics of sic nmos.

Consider the schematic diagram of SiC NMOS for the evaluation of DC transfer characteristics, as shown in Fig 7. The DC analysis was performed by fixing the drain to source voltage (*V*_*DS*_) at 20V at 25°*C* and linearly sweeping the input terminal voltage (*V*_*GS*_) from 0 to 10 V.

**Fig 8:**
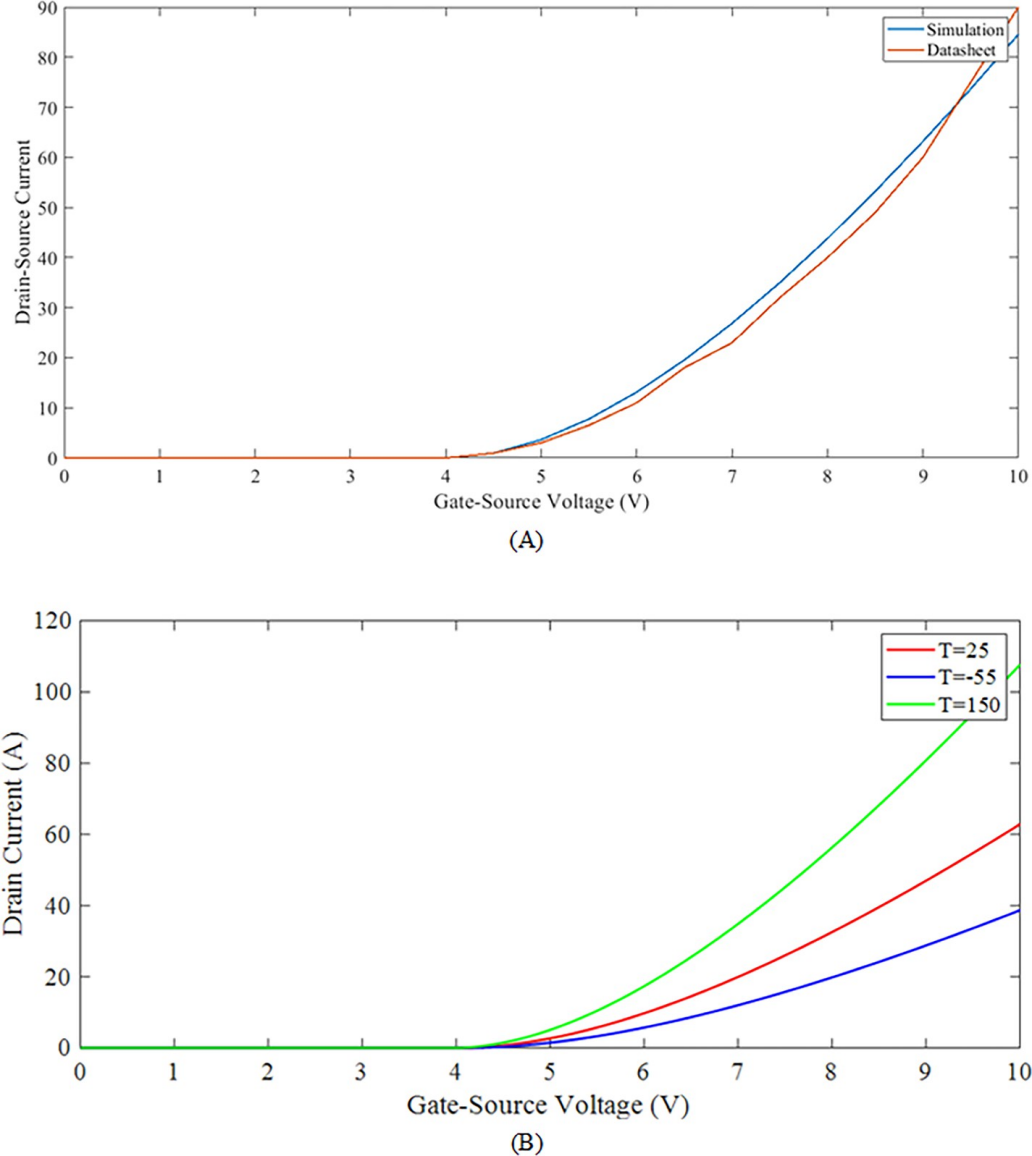
Comparison of proposed SiC MOSFET model input transfer characteristics (A) impact of temperature (B) comparison to datasheet at ***T* = 25°*C***.

As the input terminal voltage is linearly enhanced from 0 to 4 volts, initially, no current flow occurs between the drain to the source terminal and the device is observed to remain in a cut-off state, as shown in Fig 8. As the input voltage magnitude exceeds the 4 volts, a very small channel begins to form between the drain and source, and with the development of this channel, the electron flow takes place and a small magnitude of the current is observed. As the input voltage magnitude is increased further, the flow of current increases non-linearly and is observed from Fig 8A). Fig 8A represents the impact of changing temperature on the device characteristics. As the temperature is raised, the drain current drastically rises for the same amount of the drain side voltage. These results have been compared with the transfer characteristics of the SiC NMOS datasheet as well and it can be seen in Fig 8B), that the proposed model curves are very closed to the datasheet of C2M0025120D and this suggests our extracted parameters are working fine.

**Fig 9:**
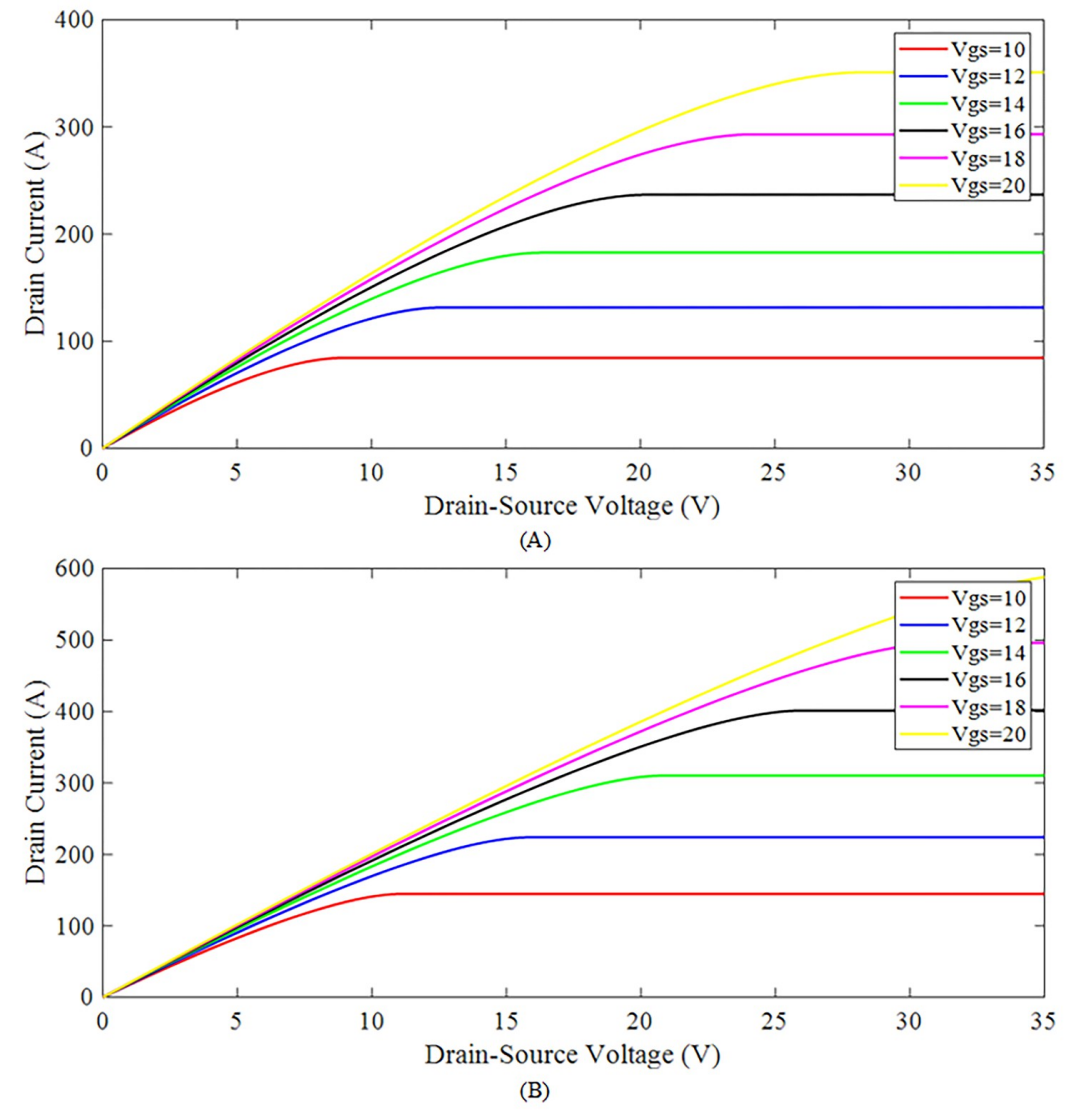
Sic nmos output transfer characteristics of the proposed model (A) at **25°*C*** (B) at **150°*C***.

The output DC transfer characteristic curves are obtained by linearly increasing the drain to source voltage (*V*_*DS*_) from 0 to 35 volts and sweeping the input terminal voltage (*V*_*GS*_) between 10 to 20 volts. It can be seen from Fig 9 that as the gate to source voltage is linearly increased in steps, the drain to source current (*I*_*DS*_) also increases. The *I*_*DS*_ linearly increase in the beginning and as the NMOS enters the saturation region, the current becomes constant. At this point, the NMOS is not connected to any other external load resistance. In this phase, the presented model is also simulated to test the parameter dependency over the temperature. In Fig 9A the analysis for the output transfer characteristic is performed at room temperature i.e. 25°*C* and the output transfer characteristic at 150°*C* is shown in Fig 9B. As the temperature is raised from 25°*C* to 150°*C*, the curves undergo a remarkable change in their drain current. By increasing the temperature, the drain current significantly rises and the curves reach the saturation region earlier at high temperature as compared to the room temperature. These results can be easily compared with that of the datasheet as given in [33].

**Fig 10:**
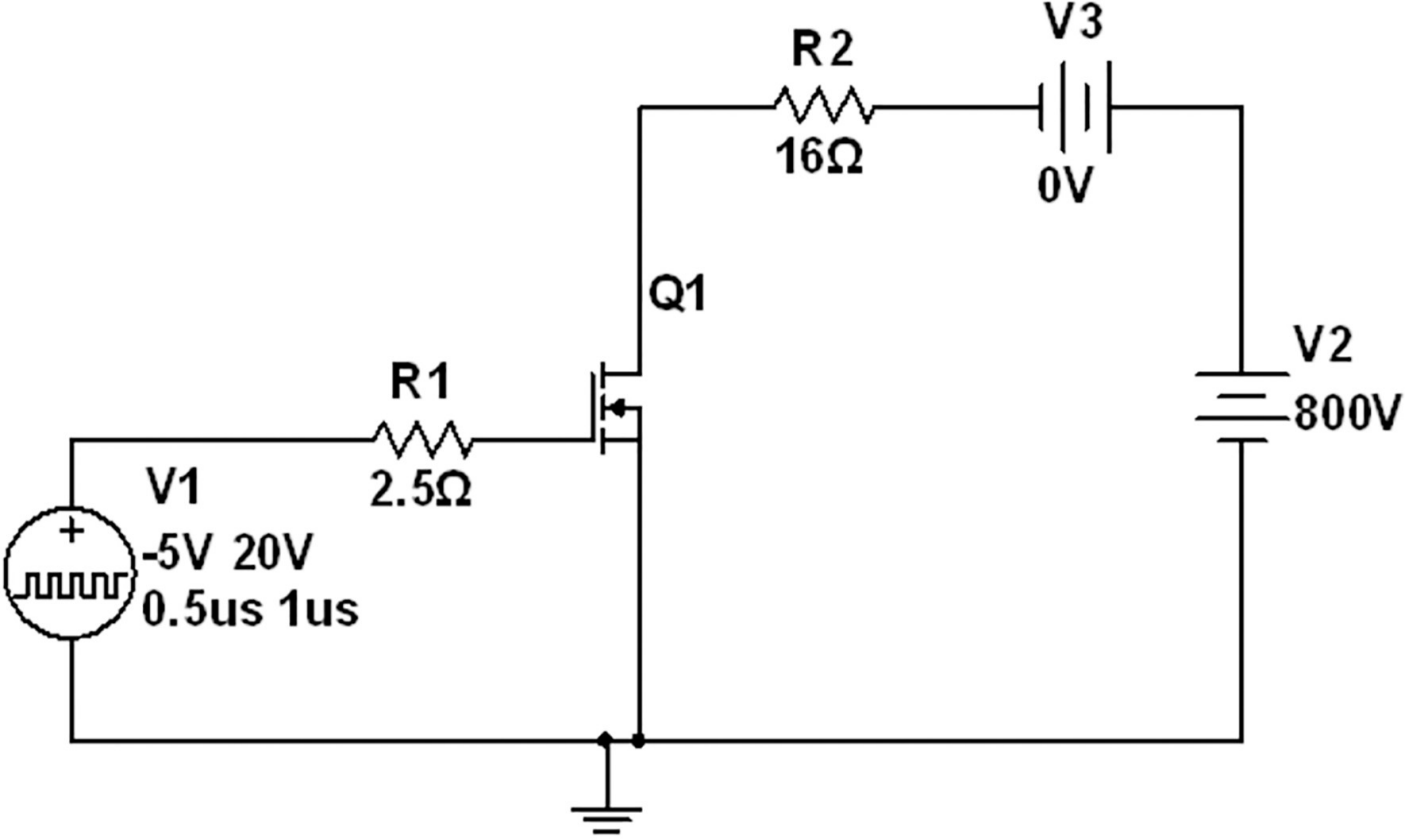
SiC nmos circuit for its transient analysis.

**Fig 11:**
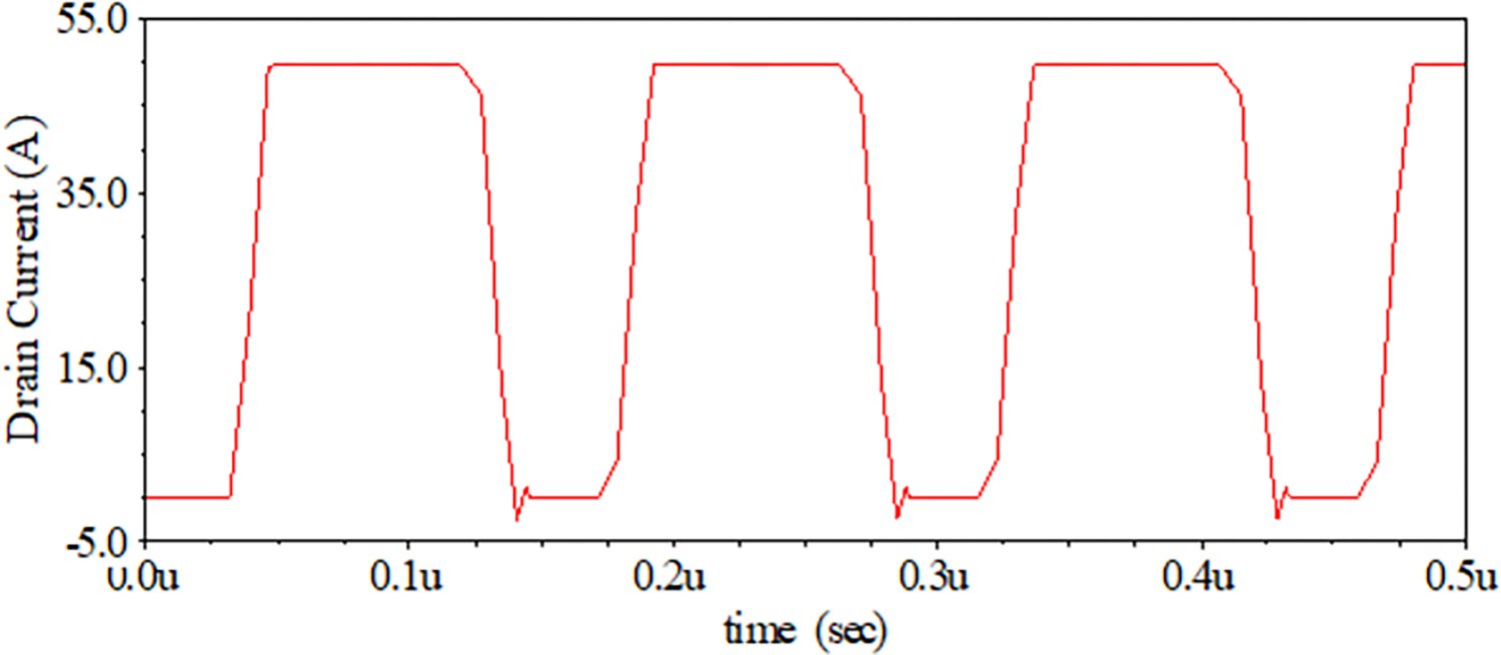
Transient analysis of proposed sic nmos for pulse (*μ* = micro).

To perform the transient analysis requires a continuous pulsating input between -5/20 Volts applied at the gate terminal. A pulsating signal with a 50% duty cycle and a 1MHz switching frequency is applied between the gate and source terminal. The gate external resistance $\left(R_{G_{ext}}\right)$ is increased linearly in steps. The drain to source voltage is fixed at 800V and a 16Ω resistive load is connected between the drain and the DC battery. The schematic diagram of the circuit is shown in Fig 10. It should be noted here that the gate internal parallel equivalent resistance is 8.55. As the gate external resistance is increased, the rise and fall time steadily rises due to the presence of the parasitic resistive components at the gate. The transient analysis for our proposed model is summarized in Table 2. In other words, with the increase in gate external resistance, the gate offers more delays. From Table 2, it can be observed that the rise time of our proposed model almost aligns with the rise time of the practical model datasheet, as shown in Fig 11.

**Table 2:** Effect of changing external resistance over device transients.

	Transients mentioned in the model datasheet (C2M002512D)		Transients offered by proposed model for extracted parameters	
$R_{g_{ext}}$	*t*_*r*_	*t*_*f*_	*t*_*r*_	*t*_*f*_
**2.5**	32	29	28	28
**4**	36	33	33	29
**8**	50	45	47	43
**12**	62	56	62	45
**16**	75	67	79	53

**Fig 12:**
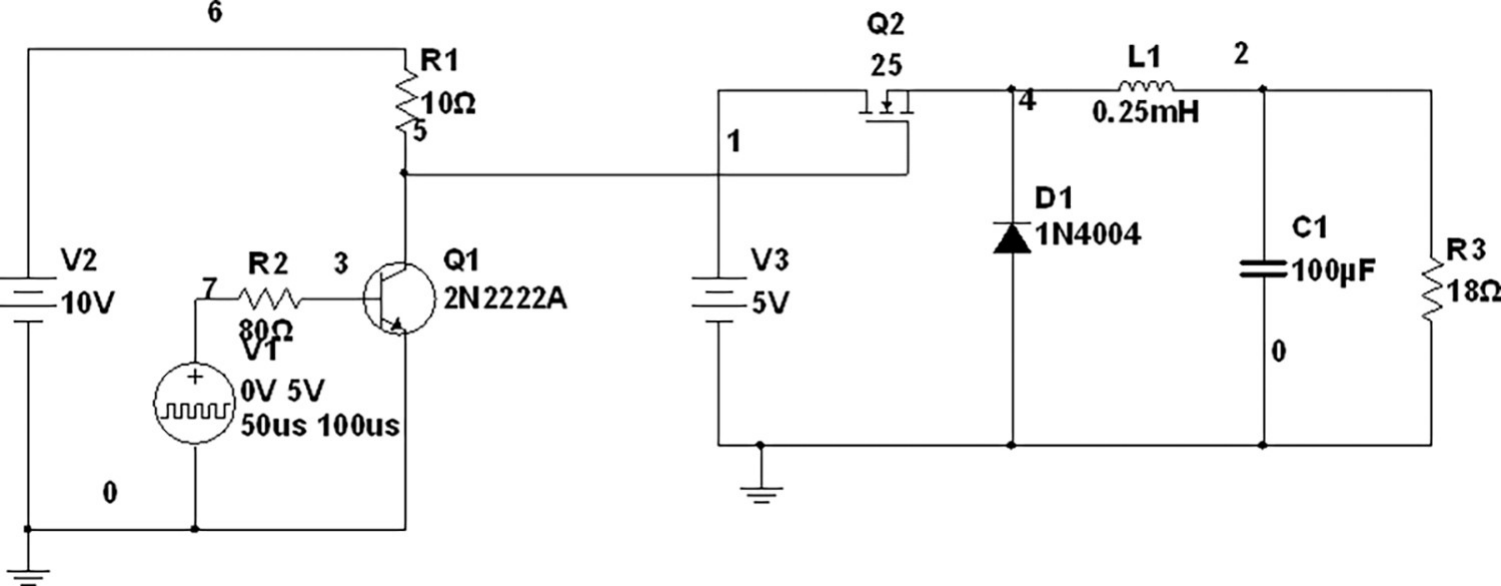
SiC nmos circuit for its transient analysis.

**Fig 13:**
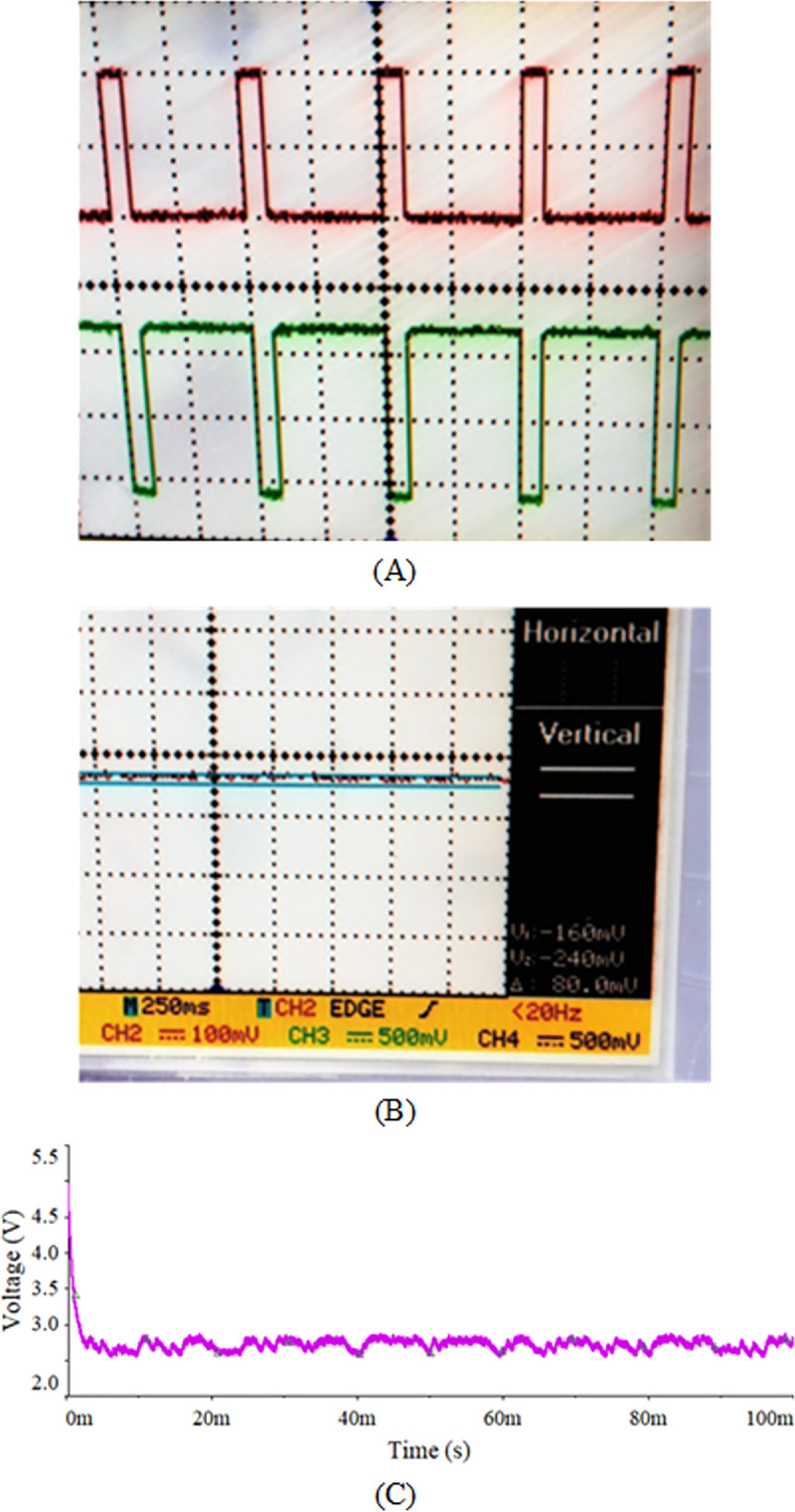
(A) PWM waveform of driving circuit on the oscilloscope, (B) output waveform of a dc-dc buck converter using the oscilloscope, and (C) output voltage of dc-dc buck converter through simulation.

**Fig 14:**
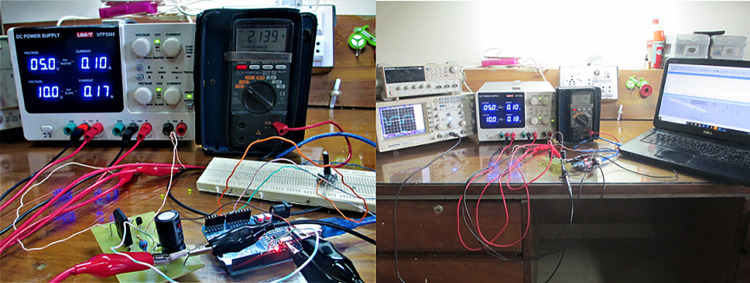
Experimental setup of sic-based buck convertor.

A small-scale lab test bench has been performed to verify the extracted parameters from our proposed techniques. The purpose of the small-scale 2:1 buck converter setup is to ensure the validity of our model by using C2M0025120D as a practical NMOS in a buck converter. These experimental results will ensure the accuracy of our extracted parameters. The components used for its implementation are summarized in Table 3. The NMOS is derived by using a 10 V input through its gate driver, as shown in the schematic diagram in Fig 12. The gate voltage and the output stepped down voltage waveforms are shown in (Fig 13A–13C) along with the complete experimental setup is shown in Fig 14. The results obtained from practical and the one used in SPICE are very closely related to each other and are summarized in Table 4. Both results are obtained against 10kHz switching frequency, same input voltage i.e. 5 volts, and at the same duty cycle. However, there is a minute difference between simulation and practical results. This is due to the surrounding temperature and tolerance in basic components. But, the overall results are satisfying.

**Table 3:** List of components used for lab testing.

Component/Equipment	Value/Model
Nmos	C2m0025120d
Diode	1n4004
Capacitor	100*μF*
Inductor	0.25*mH*
Controller	Arduino Uno
npn Transistor	2N2222
Regulated Dc Power Supply	10V, 5V
Digital Oscilloscope	GDS-2000 Series

**Table 4:** Comparison of simulation model results with that of practical lab results.

Parameters	Simulation Results	Practical Results
Input Voltage (V)	5	5
Output Voltage (V)	2.7	2.43
Ripple Voltage (mV)	53	80
Overshoot Voltage (V)	0	0
Inductor (mH)	0.25	0.25
Capacitor (***μ***f)	100	100
Operating Frequency (kHz)	10	10
Min. Load Current (mA)	36	100
Max. Load Current (mA)	150	170
Duty Cycle (%)	30	30

## Application

In a very large-scale integration (VLSI) domain, SPICE software packages are mostly used by semiconductor device manufacturer companies to optimize the device characteristics in the integration and fabrication process. Since the adaptability of renewable energy sources is highly dependent on power electronic semiconducting devices, therefore a scalable semiconductor model is often required to test its characteristics after integrating it with the required circuit. Here comes the contribution of this paper, the proposed method with which a nanoscale change can be proposed to optimize the device transfer characteristics along with high order system simulation results with great accuracy. With the help of this parameter extraction method any SiC MOSFET parameter could be extracted and can be modelled in SPICE language for circuit simulation with a high accuracy. The proposed method finds its applications in digital logic designs, digital communication, and automation systems that create a sustainable environment.

## Conclusion

The resulting characteristics curves of SiC MOSFET model are very close to the results as reported by the manufacturer data sheet. The results of programmable model while implementing a DC-DC buck converter also align with the experimental results. The experimental analysis has been performed at standard room temperature. However, the simulation results are also conducted at different temperature level to verify the effect over device electrical characteristics. The model transient analysis has also been tested by varying gate resistance and the output characteristics, which linearly aligns with the manufactured device. The programing model is developed using SPICE software. But the parameters thus extracted using proposed model can be easily integrated in other software like MATLAB and MULTISIM. The key tuning parameter during modeling is the proper selection of device process constant, improper selection will introduce error in remaing parameters. The current model is limited to SPICE LEVEL 3 and it can be further extended to higher level in future based on extracted parameters.

## Supporting information

S1 Fig Code for generating pulses for Arduino. This generates pulses for nmos used in experiment.
(TIF)
