# Supplementary figures and images for: Parameter extraction and selection for a scalable N-type SiC MOSFETs model and characteristic verification along with conventional dc-dc buck converter integration
Source: PLoS One. 2023 Jan 13;18(1):e0277331. doi: 10.1371/journal.pone.0277331 (PMC9838867; doi:10.1371/journal.pone.0277331)

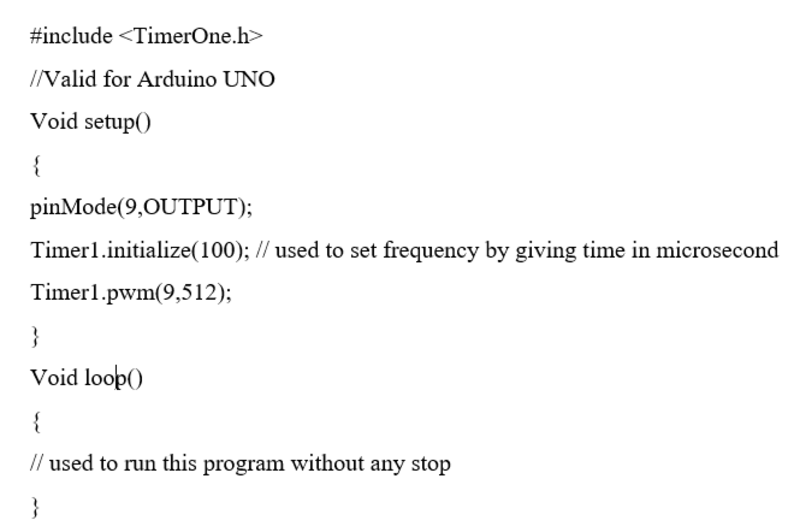

Supplement: S1 Fig — This generates pulses for nmos used in experiment. (TIF) [file pone.0277331.s001.tif]
